# Antimicrobial Susceptibility Patterns and Proportion of Multidrug‐Resistant Uropathogens in a Remote Tertiary Hospital in Bhutan: A Cross‐Sectional Study

**DOI:** 10.1002/hsr2.73364

**Published:** 2026-10-07

**Authors:** Sanga Tenzin, Yeshey Dorjey, Pelden Wangchuk, Chimi Wangmo, Nima Dorji, Ugyen Rinzin, Ugyen Chenzom, Tsheten Wangchuk

**Affiliations:** ^1^ Department of Pharmacy Regional Referral Hospital Mongar Bhutan; ^2^ Department of Obstetrics and Gynecology Regional Referral Hospital Mongar Bhutan; ^3^ Office of the Medical Superintendent Regional Referral Hospital Mongar Bhutan; ^4^ Department of Pathology and Laboratory Medicine Regional Referral Hospital Mongar Bhutan; ^5^ Department of Emergency Medicine Regional Referral Hospital Mongar Bhutan; ^6^ Department of Surgery Regional Referral Hospital Mongar Bhutan

**Keywords:** Bhutan, Escherichia coli, Klebsiella pneumoniae, multidrug‐resistance, urinary tract infections

## Abstract

**Background and Aims:**

A surge in antimicrobial resistance and multidrug‐resistant organisms among uropathogens have complicated its management over time. It is imperative to examine epidemiology and local antimicrobial susceptibility patterns to promote evidence‐based management. This study aimed to evaluate the antimicrobial susceptibility patterns of uropathogens and proportion of multidrug‐resistant organisms at a remote tertiary hospital in Bhutan.

**Methods:**

A cross‐sectional study was conducted in the Microbiology Laboratory of a remote tertiary hospital in Bhutan, by retrospectively reviewing medical records of patients who underwent urine culture and sensitivity testing from January 1 to December 31, 2025. All culture ‐ positive (≥ 10^5^ CFU/mL) samples were included in the study. Data were collected using a structured proforma and analyzed using SPSS version 23.

**Results:**

A total of 1867 urine samples were analyzed, of which 19.9% were culture positive. Culture positivity was more common in female (85.8%), and older than 40 years (55%). Gram‐negative bacteria constituted the majority (98.4%), with *E. coli* (80.6%) being the most frequently isolated pathogen, followed by *Klebsiella pneumoniae* (15.6%). *E. coli* demonstrated high sensitivity to Access group‐gentamicin (81.1%) and Watch group‐Imipenem (84.2%). Nearly two‐fifths of the uropathogen isolates were multidrug‐resistant (39.2%), and a few isolates were carbapenem‐resistant organism (8.2%). Multidrug‐resistant uropathogens were significantly reported from hospitalized patients (77.4% vs 22.6%, *p* < 0.001). On multivariable analysis, inpatient status was the only independent predictor of multidrug‐resistant organisam isolation (aOR 2.4, 95% CI 1.34–4.37, *p* = 0.003).

**Conclusion:**

Most of the culture positive were caused by Gram‐negative bacteria‐*E. coli* demonstrating high susceptibility to gentamicin. Multidrug‐resistant organisms were prevalent in nearly two‐fifths of the isolates. These findings provide baseline data to guide empirical therapy and support antimicrobial stewardship program.

**Abbreviations**
CLSI: Clinical and Laboratory Standards Institute
CRO: Carbapenem‐resistant organism
ESBL: Extended‐spectrum beta‐lactamase
ESKAPEE: *Enterococcus faecium*, *Staphylococcus aureus, Klebsiella pneumoniae, Acinetobacter baumannii, Pseudomonas aeruginosa*, *Enterobacter* species and *Escherichia coli*
IPD: Inpatient department
MDRO: Multidrug‐resistant organism
OPD: Outpatient department
p‐PDR: Possible pandrug‐resistant
p‐XDR: Possible extensively drug‐resistant
UTI: Urinary tract infection
WHO: World Health Organization

## 1 Introduction

Urinary tract infection (UTI) is an infection caused by bacteria in various parts of the urinary system which is diagnosed based on clinical signs, symptoms and further confirmed by urine culture [1]. The most common organism causing UTI worldwide is Gram‐negative bacteria *E*. coli accounting for 80%–90%, followed by *Klebsiella pneumoniae, Pseudomonas* spp.‐ while Gram‐positive bacteria *Streptococcus* spp. and *Staphylococcus* spp. accounts for small portion of infection [2, 3, 4]. A similar causative organism causing UTI were reported from low‐ and low‐middle income countries [5, 6, 7, 8, 9, 10, 11]. Uropathogenesis is determined not only by the presence of bacteria but also by the interaction between microbial virulence factors and host. Uropathogens utilize specialized fitness mechanisms and complex host–pathogen interactions – such as forming intracellular bacterial communities and modulating surface antigens – to breach mucosal innate immune defenses and evade host clearance [12] The human urinary system maintains homeostasis and fights infection through integrated innate and adaptive immune mechanisms. These include pattern‐recognition receptors like Toll‐like receptors, interleukins, macrophages, and urinary complement profiles, which work alongside nervous system signaling to regulate inflammation, detect pathogens, and support targeted strategies against recurrent urinary tract infections. Disruption of these homeostatic and immune defenses by host factors (such as age, diabetes, or behavioral risk factors) and underlying comorbidities may lead to an increased risk of infection [13]

Antibiotic treatment is considered as vital for countering any bacterial infections including UTIs [14]. However, the rising antimicrobial resistance among Gram‐negative pathogens like *E. coli* and *Klebsiella pneumoniae* – key members of the high‐priority ESKAPEE group (*Enterococcus faecium*, *Staphylococcus aureus*, *Klebsiella pneumoniae*, *Acinetobacter baumannii*, *Pseudomonas aeruginosa*, *Enterobacter* species, and *E. coli*) – poses significant challenges in treating common infections. These multi‐ and extensively drug‐resistant “superbugs” have acquired defense mechanisms against multiple antimicrobial classes, drastically limiting therapeutic options and raising the risk of treatment failure [15, 16]. The resistant rate to first‐line antibiotics such as sulfamethoxazole‐trimethoprim, ciprofloxacin and ceftriaxone ranges from 33% to 90% in South East Asian region [6, 7, 9, 11, 17]. Moreover, several studies have reported rate of multidrug‐resistance UTIs ranging from 35% to 55% which further complicates its management [18, 19, 20].

Despite Antimicrobial Stewardship Programs and national antimicrobial resistance surveillance being prioritized in public health policy in Bhutan, there are significant operational gaps. In many remote healthcare settings, routine clinical practice continues to rely on empirical antimicrobial prescribing in the absence of microbiological support and local susceptibility data. This practice creates continuous selective pressure through broad‐spectrum empirical therapy, thereby undermining broader stewardship objectives across the health system. To our knowledge, there are limited published data on antimicrobial susceptibility patterns from Bhutan and especially in this remote tertiary hospital which serves as the main referral centre for the eastern districts, catering to a diverse population across the region. While this study relies on conventional microbiological surveillance methods rather than novel molecular techniques, it provides critical baseline data from a geographically underrepresented region in eastern Bhutan, which plays a critical role for guiding empirical treatment, improving patient outcomes and supporting antimicrobial stewardship programs. Therefore, this study aimed to describe the antimicrobial susceptibility patterns and the proportion of multidrug‐resistant uropathogens.

## 2 Materials and Methods

### 2.1 Study Design and Setting

A cross‐sectional study was conducted at a remote tertiary hospital in Bhutan, by retrospectively reviewing records at the Microbiology Laboratory from January 1 to December 31, 2025. Bhutan is a landlocked nation nestled in the Eastern Himalayas between China and India with population of over 0.78 million [21]. Bhutan's healthcare system is a predominantly government funded and follows a three‐tier structure which consists of primary level (primary healthcare centers, sub‐posts and outreach clinics), secondary level (cluster and district hospital) and tertiary level (national and regional referral hospital) [22]. This hospital is one of the three tertiary hospitals. It serves as a main medical center for the eastern districts. The hospital has 150 beds and offers a wide range of specialized clinical services, including Internal Medicine, Emergency Medicine, General Surgery, Obstetrics & Gynecology, Pediatrics, Orthopedics, Ophthalmology, Otolaryngology, Pathology & Laboratory Medicine, Pharmacy and Dental Surgery, besides other services. This hospital is equipped with diagnostic facilities through its Department of Pathology & Laboratory Medicine. Diagnostic services include hematology, biochemistry, blood banking, histopathology and microbiology. The Microbiology Laboratory provides culture and susceptibility testing for various clinical specimens such as blood, urine, cerebrospinal fluid, pus, urethral swab, vaginal discharge, body fluid, tissue specimen and sputum.

### 2.2 Study Population

All patients whose urine sample were submitted for culture and susceptibility testing at Microbiology Laboratory in the year 2025 were reviewed and those with positive urine culture (≥ 10^5^ CFU/mL) were included in the study. Those samples with negative culture, polymicrobial growth, duplicate isolates, coagulase‐negative Staphylococcus (CoNS), and incomplete data were excluded from the study. However, during data extraction, there were no polymicrobial growth, duplicate isolates, or incomplete records. There were 372 positive cultures during the year 2025‐which was included for study, and a separate sample size was not calculated. Data were collected using a consecutive sampling method.

### 2.3 Study Procedure

Laboratory register maintained at Microbiology Laboratory was retrospectively reviewed and the data were extracted using a structured proforma developed for the study. Demographic characteristics (age, gender), hospital departments, hospital visit types [Inpatient department (IPD), and outpatient department (OPD)], microbial isolates, and patterns of antimicrobial susceptibility of culture (susceptible, intermediate and resistant), multidrug‐resistant organisms, and carbapenem‐resistant organisms were all collected.

### 2.4 Microbiological Method

Urine specimens were collected following the standard operating procedures where clean – catch midstream urine were collected in sterile, wide‐mouth containers after providing explicit verbal instructions on periurethral cleansing to minimize skin and commensal flora contamination for non‐catheterized patients and for catheterized patients, urine specimens were obtained via aseptic needle aspiration from the sampling port of the indwelling catheter system following transient clamping of the tubing. These specimens were processed immediately upon receipt or within 2 h in the microbiology laboratory. Each specimen was mixed thoroughly before inoculation onto Cystine Lactose Electrolyte Deficient (CLED) agar using a sterile calibrated 1‐µL inoculating loop following standard semi‐quantitative plating technique. Culture plates were incubated aerobically at 37°C for 18–24 h. Following incubation, culture plates were examined for bacterial growth, colony morphology, lactose fermentation, and evidence of mixed growth suggestive of contamination. Colony counts were expressed as colony‐forming units per milliliter (CFU/mL) based on the calibrated loop volume. Growth of ≥ 10^5^ CFU/mL was considered significant bacteriuria, and growth of 10^4^– < 10^5^ CFU/mL reported as probably significant, whereas < 10^4^ CFU/mL was reported as insignificant growth. Significant bacterial isolates were identified using conventional microbiological methods, including assessment of colony morphology, gram staining and standard biochemical tests. Isolates requiring further confirmation were identified using the Analytical Profile Index identification system. Antimicrobial susceptibility testing was performed using the Kirby–Bauer disk diffusion method and zone diameter results were interpreted using the breakpoints specified in the Clinical and Laboratory Standards Institute (CLSI) Performance Standards for Antimicrobial Susceptibility Testing (M100, 33rd Edition, 2023). Isolates were categorized as susceptible, intermediate, or resistant, with intermediate results reported as a standalone category in the primary tabular data. For visual presentation and drug‐class analysis, isolates were classified into binary categories: Susceptible (S) and Non‐Susceptible (NS), where Non‐Susceptible (NS) represents a pooled analytical category combining Intermediate (I) and Resistant (R) strains. Antimicrobial susceptibility testing followed a tiered approach, where second‐line agents were evaluated only when isolates demonstrated non‐susceptibility (intermediate or resistant) to first‐line drugs. As a result, susceptibility rates for second‐tier antibiotics reflect conditional probabilities subject to selection bias, as testing was preferentially performed on a pre‐selected subset of resistant isolates. Quality control was performed using standard reference strains, including *Escherichia coli* ATCC 25922, *Staphylococcus aureus* ATCC 25923, and *Pseudomonas aeruginosa* ATCC 27853.

### 2.5 Operational Definitions

**Multidrug‐resistant organism (MDRO):** Antimicrobial resistance profiles were categorized based on international consensus definitions established by the European Centre for Disease Prevention and Control (ECDC) and Centres for Diease Control and Prevention (CDC) [23]. A multidrug‐resistant organism (MDRO) was defined as an isolate demonstrating acquired non‐susceptibility (i.e., intermediate or resistant) to at least one agent in three or more antimicrobial categories. Extensively drug‐resistant (XDR**)** was defined as non‐susceptibility to at least one agent in all but two or fewer antimicrobial categories (i.e., isolates remaining susceptible to only one or two categories), and pandrug‐resistant (PDR) was defined as non‐susceptibility to all tested agents across all antimicrobial categories. As per international consensus guidelines, in cases of incomplete testing where all recommended drug categories are not evaluated, isolates can only be characterized as possible XDR (p‐XDR) or possible PDR (p‐PDR) [23]. Because only seven antimicrobial classes were available in our laboratory panel, we adopted these consensus provisions, designating isolates showing non‐susceptibility to all but ≤ 2 tested classes as p‐XDR, and those non‐susceptible across all seven available classes as p‐PDR.

**Carbapenem‐resistant organism (CRO):** are the bacteria that are resistant to at least one of the carbapenem class of antibiotics [24].

**WHO AWaRe classification of antibiotics**‐ to assist in the development of tools for antibiotics stewardship program, and to reduce antibiotic resistance, AWaRe classification of antibiotics (access, watch, and reserve) was developed by World Health Organization (WHO). **Access group** includes antibiotics that have activity against a wide range of commonly encountered susceptible pathogens while also showing lower resistance potential than antibiotics in the other groups. **Watch group** includes antibiotics that have higher resistance potential and includes most of the highest priority agents among the critically important antimicrobials. **Reserve group** includes antibiotics that should be reserved for treatment of confirmed or suspected infections due to MDRO. These antibiotics should be treated as “last resort” options [25].

### 2.6 Data Management and Confidentiality

A unique ID was assigned to each patient included in the study, and the investigators carefully entered data in REDCap and the data was double checked for any duplication. The principal investigator supervised the data collection procedure and reviewed the collected data form for completeness. Confidentiality was maintained by keeping the data in a secure folder locked with a password. The questionnaire did not include any identifying information.

### 2.7 Data Analysis

Data were captured using REDCap‐exported and analyzed using SPSS Version 23 (IBM Corp., Armonk, NY, USA). The Kolmogorov‐Smirnov test was used to assess the normality of continuous variables. Age was not normally distributed and compared between the OPD and IPD using Mann‐Whitney U test and expressed as median (IQR). Categorical variables were analyzed using the Chi‐square test or Fisher's exact test, as appropriate, and presented as frequencies and percentages, and a *p*‐value < 0.05 was considered statistically significant. Variables with *p* < 0.2 on univariate analysis were entered into a multivariable logistic regression model, with MDRO status (present/absent) as the binary outcome variable, to identify independent predictors of MDRO isolation. Model fit was assessed using the Hosmer‐Lemeshow goodness‐of‐fit test, and multicollinearity among independent variables was evaluated using variance inflation factors (VIF), with VIF > 5 considered indicative of significant multicollinearity. Results were expressed as crude and adjusted odds ratios (cOR/aOR) with 95% confidence intervals (CI).

## 3 Results

In 2025, 1867 patients underwent urine culture and susceptibility testing, of whom approximately one‐fifth (19.9%, 372/1867) were culture positive. Among these, around 60% (222/372) were from the IPD while the remaining 40% (150/372) from the OPD.

### 3.1 Departments and Demographic Characteristics

More than half (55.4%, 206/372) of the patients were over 40 years of age, and positive cultures were significantly higher in females than in males (85.8% vs 14.2%, *p* < 0.001). The median age of IPD patients were significantly higher compared to OPD patients [52 (31.5–74.0) vs 37 (24.0–48.2); *p* < 0.001)]. Across age categories, *E. coli* was the predominant uropathogen (83.9% in < 21 years, 80.0% in 21–40 years, and 77.7% in > 40 years), followed by *Klebsiella pneumoniae* (16.1% in < 21 years, 13.6% in 21–40 years, and 16.0% in > 40 years), with no statistically significant variation across age intervals (*p *= 0.590) (Supplementary Table 2). Similarly, *E. coli* predominated across both sex cohorts (80.9% in females vs. 69.8% in males), while *K. pneumoniae* accounted for 14.4% of isolates in females and 20.8% in males, showing no statistically significant differences by gender (*p *= 0.145) (Supplementary Table 3). The overall highest proportion of positive cultures were observed among patients from the General Practice Department (30.9%), followed by the Emergency Department (25.8%). Detailed distributions are presented in Table 1.

**Table 1:** Departments and demographic characteristics of patients with positive urine culture at the remote Tertiary Hospital, Bhutan (*n* = 372).

Characteristics	Total	OPD	IPD	*p*‐value
**Age, *n* (%)**				
< 21 years	56 (15.1)	29 (19.3)	27 (12.2)	< 0.001^a^
21–40 years	110 (29.6)	58 (38.7)	52 (23.4)	
> 40 years	206 (55.4)	63 (42.0)	143 (64.4)	
**Median age (IQR)**	45 (28.2–66.0)	37 (24.0–48.2)	52 (31.5–74.0)	< 0.001^b^
**Sex, *n* (%)**				
Female	319 (85.8)	142 (94.7)	177 (79.7)	< 0.001^a^
Male	53 (14.2)	8 (5.3)	45 (20.3)	
**Departments, *n* (%)**				
Emergency	96 (25.8)	0 (0.0)	96 (43.2)	< 0.001^a^
General practice	115 (30.9)	80 (53.3)	35 (15.8)	
Obstetrics and gynecology	60 (16.1)	40 (26.7)	20 (9.0)	
Medicine	37 (9.9)	2 (1.3)	35 (15.8)	
Pediatrics	34 (9.1)	19 (12.7)	15 (6.8)	
Surgery	30 (8.1)	9 (6.0)	21 (9.5)	

Abbreviations: IPD, inpatient department; IQR, interquartile range; OPD, outpatient department.

^a^ Chi‐square test.

^b^ Mann Whitney U test.

### 3.2 Uropathogenic Isolate Distribution

Majority of positive culture were contributed by Gram‐negative uropathogens (98.4%, 366/372), of which approximately 81% (295/366) were due to *E. coli*, followed by *Klebsiella pneumoniae* (15.6%, 57/366). In contrast, Gram‐positive organisms were minimal (Table 2). Overall, *E. coli* represented 79.3% (295/372) of total isolates across the entire study population The overall species distribution relative to the total population (N = 372) is illustrated in Figure 1.

**Table 2:** Uropathogen isolates of the patients with positive urine culture at the remote Tertiary Hospital, Bhutan (*n* = 372).

Uropathogen isolates	Total	OPD	IPD	*p*‐value
**Gram stain, *n* (%)**				
Negative	366 (98.4)	147 (98.0)	219 (98.6)	0.626
Positive	6 (1.6)	3 (2.0)	3 (1.4)	
**Gram‐negative, *n* (%)**				
*E. coli*	295 (80.6)	130 (88.4)	165 (75.3)	0.004
*Klebsiella pneumoniae*	57 (15.6)	16 (10.9)	41 (18.7)	
*Citrobacter* spp.	2 (0.5)	0 (0.0)	2 (0.9)	
*Proteus spp*.	1 (0.3)	1 (0.7)	0 (0.0)	
*Pseudomonas* spp.	11 (3.0)	0 (0.0)	11 (5.0)	
**Gram‐positive, *n* (%)**				
*Staphylococcus saprophyticus*	3 (50.0)	3 (100.0)	0 (0.0)	0.014
*Streptococcus* spp.	3 (50.0)	0 (0.0)	3 (100.0)	

Abbreviations: IPD, inpatient department; OPD, outpatient department.

**Figure 1:**
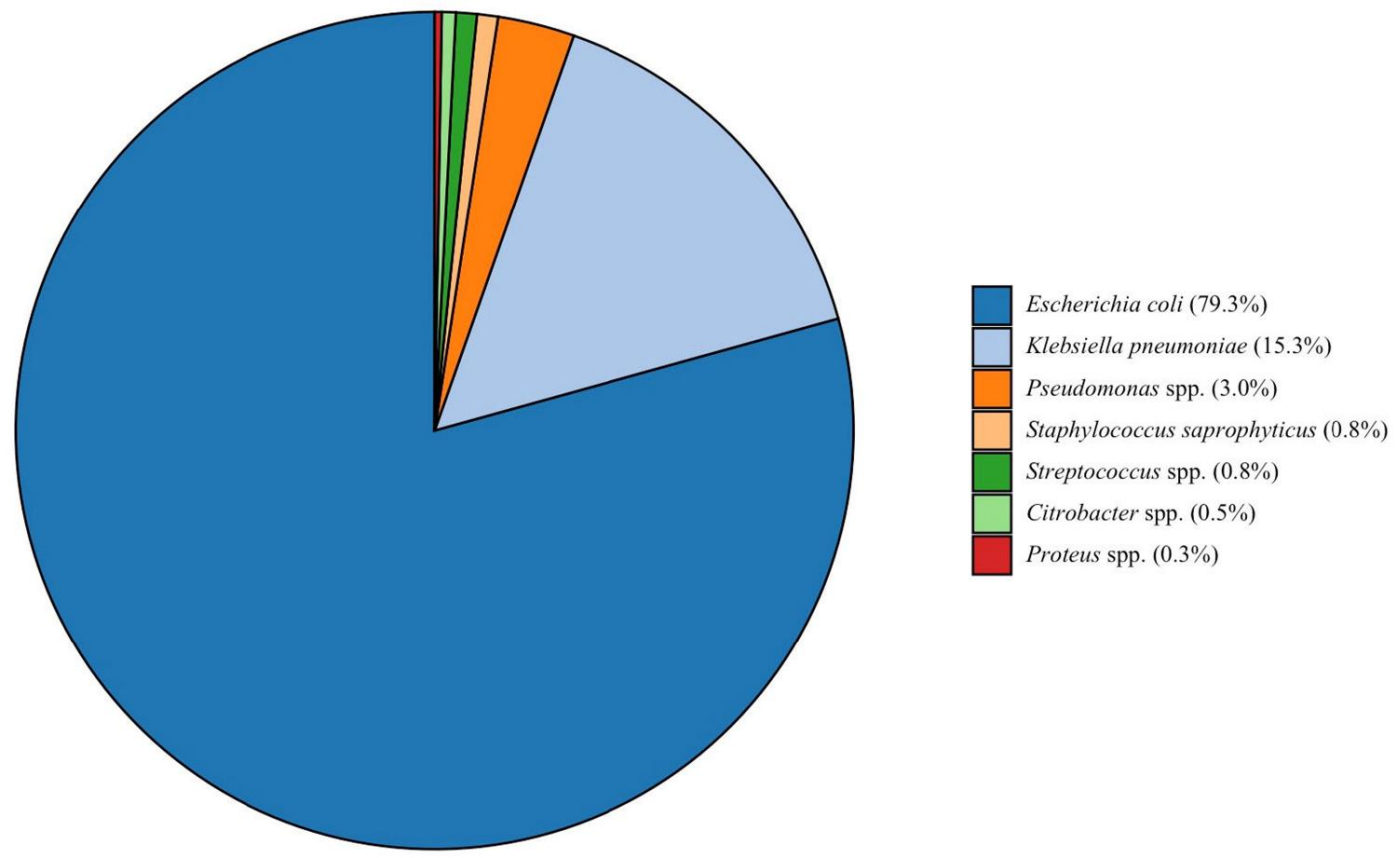
Distribution of uropathogens among culture postive patients (*N* = 372).

### 3.3 Antibiotic Susceptibility Patterns of Uropathogens

Among the isolated uropathogens, *E. coli* demonstrated high susceptibility to Access group–gentamicin (81.1%, 236/291) and amikacin (78.3%, 18/23)—as well as Watch group‐carbapenems including imipenem (84.2%, 16/19) and meropenem (75.0%, 18/24) (Figure 2). *Klebsiella pneumoniae* showed the highest susceptibility to Access group‐gentamicin (94.4%, 51/54), followed by Watch group‐norfloxacin (85.2%, 46/54) (Figure 3). The complete antibiotic susceptibility patterns are summarized in Table 3. When categorized by antibiotic drug class, aminoglycosides and carbapenems maintained high overall activity across major Gram‐negative isolates, whereas penicillins and first‐generation cephalosporins showed high rates of non‐susceptibility. Detailed susceptibility profiles across all drug classes and isolated organisms are provided in Supplementary Table 4. Across care settings, there was notable variations in susceptibility between OPD and IPD. OPD isolates demonstrated higher susceptibility to Access group antibiotics‐ ampicillin and cefazolin. In contrast, IPD isolates exhibited a higher resistance to Watch group antibiotics‐ ceftriaxone and norfloxacin. A complete breakdown of antibiotic susceptibility comparison between outpatient and inpatient settings is provided in Supplementary Table 1.

**Figure 2:**
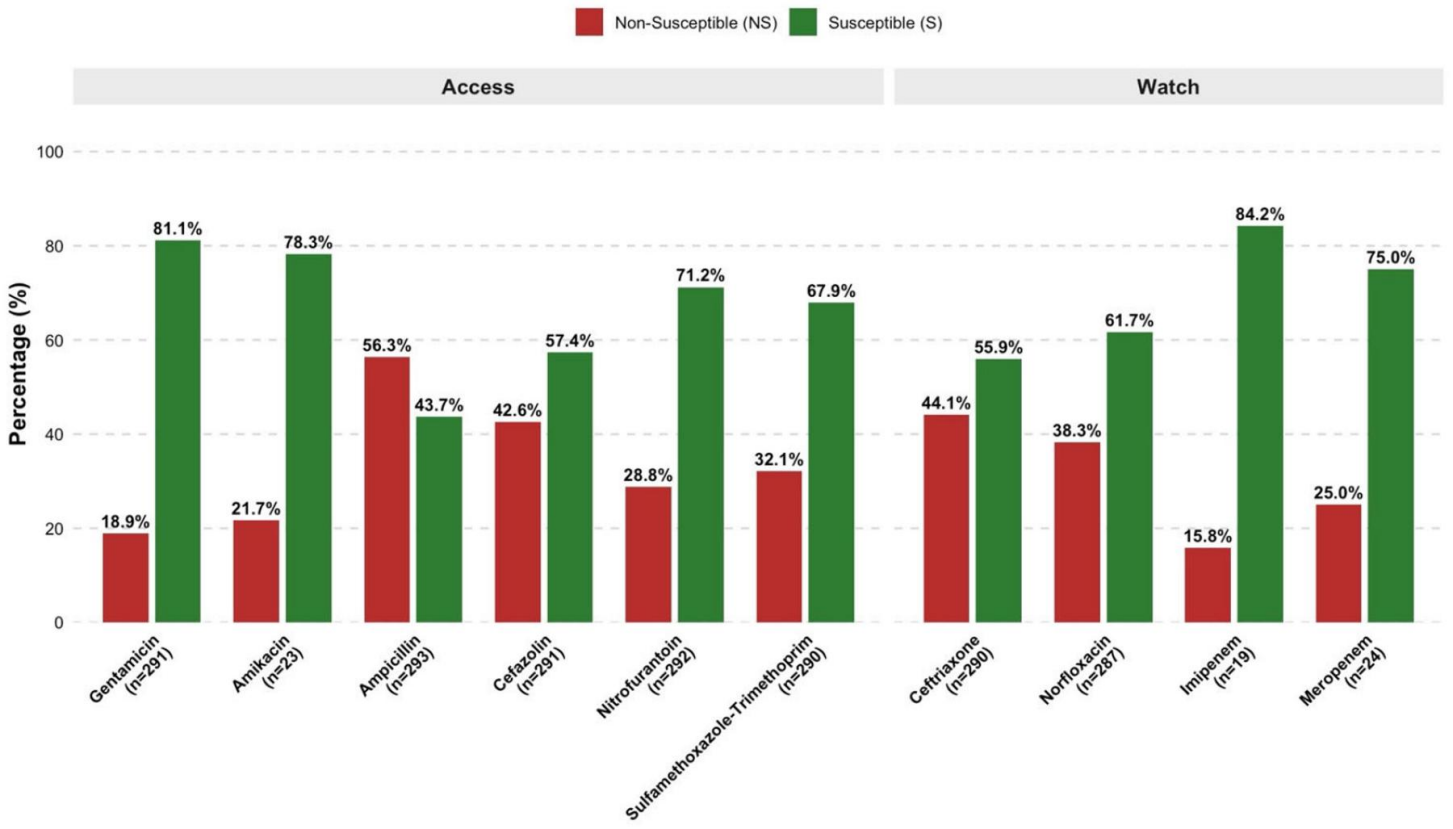
Antimicrobial susceptibility profile of *Escherichia coli* isolates across tested antimicrobials at the remote Tertiary Hospital, Bhutan. *NS represents a pooled analytical category* (*Intermediate + Resistant*)*. ‘n' values on the x‐axis represent the total denominator of isolates tested for that specific antimicrobial agent*.

**Figure 3:**
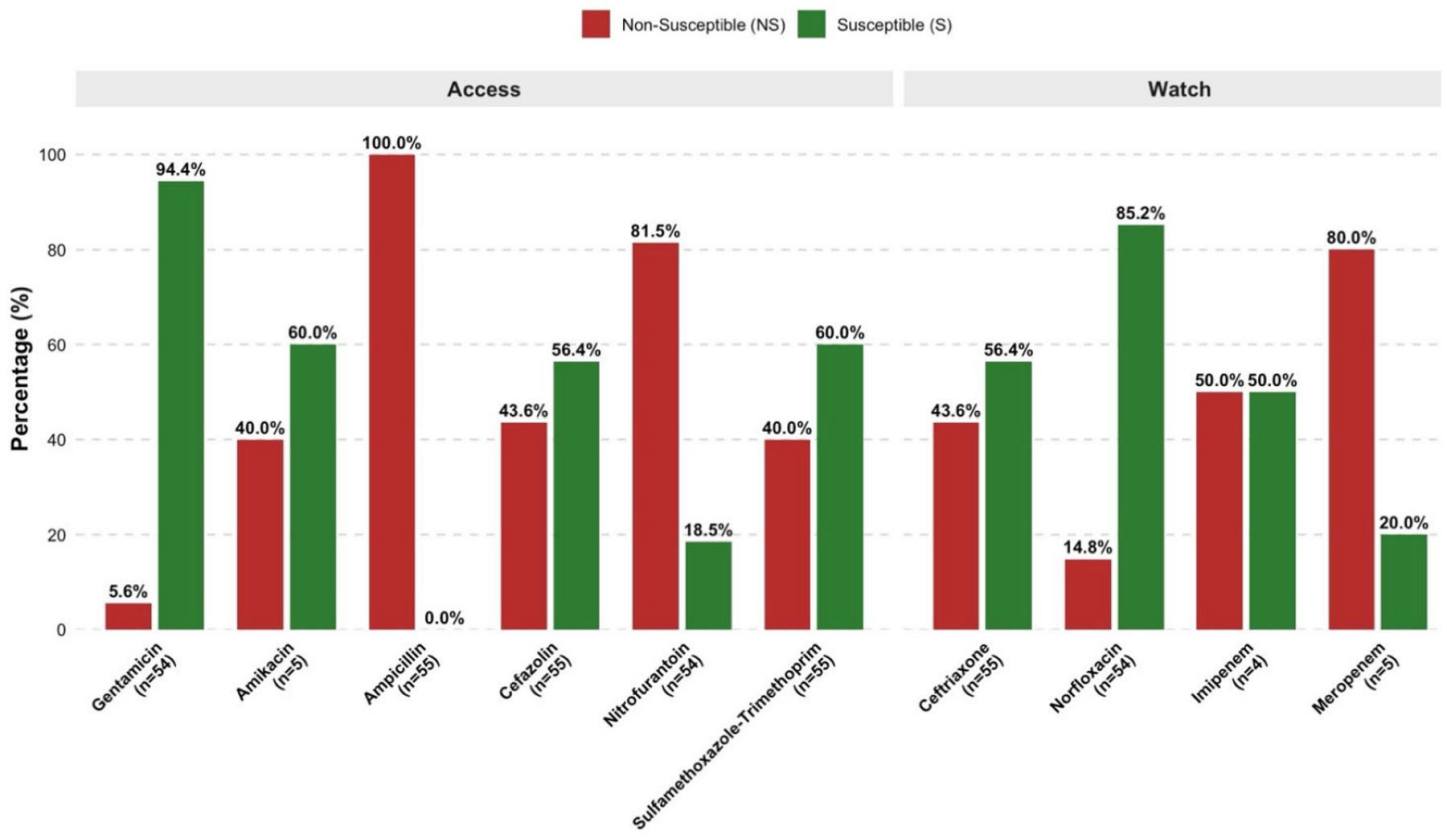
Antimicrobial susceptibility profile of *Klebsiella pneumoniae* isolates across tested antimicrobials at the remote Tertiary Hospital, Bhutan. NS represents a pooled analytical category (Intermediate + Resistant). ‘n' values on the x‐axis represent the total denominator of isolates tested for that specific antimicrobial agent.

**Table 3:** Antimicrobial susceptibility patterns of uropathogens of the patients with positive urine culture at the remote Tertiary Hospital, Bhutan (*n* = 372).

Antibiotics		*E. coli n*/*N*	*Klebsiella pneumoniae n*/*N*	*Pseudomonas* spp. *n*/*N*	*Citrobacter* spp. *n*/*N*	*Proteus* spp. *n*/*N*	*Staphylococcus saprophyticus n*/*N*	*Streptococcus* spp. *n*/*N*
Access group								
**Gentamicin (10 mcg)**								
	S	236/291 (81.1)	51/54 (94.4)	9/11 (81.8)	2/2 (100.0)	1/1 (100.0)	x	x
	I	7/291 (2.4)	0/54 (0.0)	0/11 (0.0)	0/2 (0.0)	0/1 (0.0)	x	x
	R	48/291 (16.5)	3/54 (5.6)	2/11 (18.2)	0/2 (0.0)	0/1 (0.0)	x	x
**Amikacin (30 mcg)**								
	S	18/23 (78.3)	3/5 (60.0)	5/6 (83.3)	x	x	x	x
	I	2/23 (8.7)	0/5 (0.0)	0/6 (0.0)	x	x	x	x
	R	3/23 (13.0)	2/5 (40.0)	1/6 (16.7)	x	x	x	x
**Nitrofurantoin (300 mcg)**								
	S	208/292 (71.2)	10/54 (18.5)	x	1/2 (50.0)	x	2/3 (66.7)	x
	I	28/292 (9.6)	4/54 (7.4)	x	0/2 (0.0)	x	0/3 (0.0)	x
	R	56/292 (19.2)	40/54 (74.1)	x	1/2 (50.0)	x	1/3 (33.3)	x
**Sulfamethoxazole‐trimethoprim (1.24/23.75 mcg)**								
	S	197/290 (67.9)	33/55 (60.0)	x	1/2 (50.0)	x	3/3 (100.0)	x
	I	1/290 (0.3)	2/55 (3.6)	x	0/2 (0.0)	x	0/3 (0.0)	x
	R	92/290 (31.7)	20/55 (36.4)	x	1/2 (50.0)	x	0/3 (0.0)	x
**Cefazolin (30 mcg)**								
	S	167/291 (57.4)	31/55 (56.4)	x	0/2 (0.0)	1/1 (100.0)	x	x
	I	7/291 (2.4)	1/55 (1.8)	x	0/2 (0.0)	0/1 (0.0)	x	x
	R	117/291 (40.2)	23/55 (41.8)	x	2/2 (100)	0/1 (0.0)	x	x
**Ampicillin (10 mcg)**								
	S	128/293 (43.7)	0/55 (0.0)	x	0/2 (0.0)	1/1 (100.0)	x	x
	I	8/293 (2.7)	0/55 (0.0)	x	0/2 (0.0)	0/1 (0.0)	x	x
	R	157/293 (53.6)	55/55 (100)	x	2/2 (100)	0/1 (0.0)	x	x
**Penicillin G (10 units)**								
	S	x	x	x	x	x	1/3 (33.3)	3/3 (100)
	I	x	x	x	x	x	1/3 (33.3)	0/3 (0.0)
	R	x	x	x	x	x	1/3 (33.3)	0/3 (0.0)
**Watch group**								
**Imipenem (10 mcg)**								
	S	16/19 (84.2)	2/4 (50.0)	0/1 (0.0)	x	x	x	x
	I	1/19 (5.3)	1/4 (25.0)	0/1 (0.0)	x	x	x	x
	R	2/19 (10.5)	1/4 (25.0)	1/1 (100.0)	x	x	x	x
**Meropenem (10 mcg)**								
	S	18/24 (75.0)	1/5 (20.0)	0/1 (0.0)	x	x	x	x
	I	0/24 (0.0)	0/5 (0.0)	0/1 (0.0)	x	x	x	x
	R	6/24 (25.0)	4/5 (80.0)	1/1 (100.0)	x	x	x	x
**Norfloxacin (10 mcg)**								
	S	177/287 (61.7)	46/54 (85.2)	9/10 (90.0)	1/2 (50.0)	1/1 (100.0)	3/3 (100.0)	x
	I	5/287 (1.7)	0/54 (0.0)	0/10 (0.0)	0/2 (0.0)	0/1 (0.0)	0/3 (0.0)	x
	R	105/287 (36.6)	8/54 (14.8)	1/10 (10.0)	1/2 (50.0)	0/1 (0.0)	0/3 (0.0)	x
**Ceftriaxone (30 mcg)**								
	S	162/290 (55.9)	31/55 (56.4)	x	1/2 (50.0)	1/1 (100.0)	x	x
	I	4/290 (1.4)	2/55 (3.6)	x	0/2 (0.0)	0/1 (0.0)	x	x
	R	124/290 (42.8)	22/55 (40.0)	x	1/2 (50.0)	0/1 (0.0)	x	x
**Ceftazidime (30 mcg)**								
	S	x	x	5/7 (71.4)	x	x	x	x
	I	x	x	0/7 (0.0)	x	x	x	x
	R	x	x	2/7 (28.6)	x	x	x	x
**Piperacillin (100 mcg)**								
	S	x	x	7/9 (77.8)	x	x	x	x
	I	x	x	0/9 (0.0)	x	x	x	x
	R	x	x	2/9 (22.2)	x	x	x	x

*Note:* I: intermediate; R: resistant; S: susceptible; x: not performed; n/N: ‘n'‐ represents the exact count of isolates categorized as S, I, R and ‘N'‐ represents the total number of isolates tested for that specific antibiotic‐organism.

### 3.4 Proportion of Multidrug, Extensive, and Pandrug Resistance among Uropathogens

As shown in Table 4, of the 372 patients with positive urine cultures, 39.2% (146/372) were multidrug‐resistant organisms (MDRO). Among these 146 MDRO cases, 8.2% (12/146) were carbapenem‐resistant organisms (CRO), 47.3% (69/146) met criteria for p‐XDR, and 6.8% (10/146) met criteria for p‐PDR. Inpatient department was significantly associated with MDRO status (77.4% vs 22.6%, *p* < 0.001), while all CRO (100.0%, 12/12) and p‐PDR cases (100.0%, 10/10) originated exclusively from the IPD. Across hospital departments, the Emergency Department accounted for the highest proportion of advanced phenotypes (33.6% MDRO, 50.0% CRO, 31.9% p‐XDR, and 50.0% p‐PDR). *Escherichia coli* was the predominant pathogen across MDRO (87.0%), CRO (50.0%), p‐XDR (81.2%), and p‐PDR (60.0%) categories, followed by *Klebsiella pneumoniae* (11.0% MDRO, 41.7% CRO, 17.4% p‐XDR, and 40.0% p‐PDR).

**Table 4:** Multidrug‐resistant organisms and carbapenem‐resistant organisms reported among the patients with urine culture positive at the Tertiary Hospital, Bhutan.

ariables	MDRO *n* = 146 (%)	CRO *n* = 12 (%)	p‐XDR *n* = 69 (%)	p‐PDR *n* = 10 (%)
**Visit types**				
OPD	33 (22.6)^a^	0 (0.0)	17 (24.6)	0 (0.0)
IPD	113 (77.4)	12 (100.0)	52 (75.4)	10 (100.0)
**Age (years)**				
< 21	13 (8.9)	0 (0.0)	2 (2.9)	0 (0.0)
21–40	37 (25.3)	1 (8.3)	20 (29.0)	1 (10.0)
> 40	96 (65.8)	11 (91.7)	47 (68.1)	9 (90.0)
**Gender**				
Female	116 (79.5)	5 (41.7)	55 (79.7)	5 (50.0)
Male	30 (20.5)	7 (58.3)	14 (20.3)	5 (50.0)
**Departments**				
Emergency	49 (33.6)	6 (50.0)	22 (31.9)	5 (50.0)
General practice	29 (19.9)	0 (0.0)	10 (14.5)	0 (0.0)
Obstetrics and Gynecology	19 (13.0)	1 (8.3)	15 (21.7)	1 (10.0)
Medicine	25 (17.1)	3 (25.0)	13 (18.8)	3 (30.0)
Pediatrics	8 (5.5)	0 (0.0)	1 (1.4)	0 (0.0)
Surgery	16 (11.0)	2 (16.7)	8 (11.6)	1 (10.0)
**Uropathogens**				
*E. coli*	127 (87.0)	6 (50.0)	56 (81.2)	6 (60.0)
*Klebsiella pneumoniae*	16 (11.0)	5 (41.7)	12 (17.4)	4 (40.0)
*Citrobacter* spp.	1 (0.7)	0 (0.0)	1 (1.4)	0 (0.0)
*Pseudomonas* spp.	2 (1.4)	1 (8.3)	0 (0.0)	0 (0.0)

*Note:* CRO: carbapenem‐resistant organisms; IPD: inpatient department; MDRO: multidrug‐resistant organisms; OPD: outpatient department; p‐PDR: possible – pandrug‐ resistant; p‐XDR: possible‐ extensively drug‐resistant. ^a^Chi‐square.

### 3.5 Factors Associated with MDRO

On multivariable logistic regression analysis, after adjusting for age, sex, and department, IPD status remained the only independent predictor significantly associated with multidrug‐resistant organism isolation (aOR 2.4, 95% CI 1.34–4.37, *p* = 0.003). No other variables retained statistical significance in the adjusted model. The model demonstrated good fit (Hosmer‐Lemeshow χ^2^ = 3.27, df = 8, *p* = 0.916), and no significant multicollinearity was detected among the independent variables (VIF range 1.107–1.132) (Table 5).

**Table 5:** Factors associated with multidrug‐resistant organisms among the patients with urine culture positive at the Tertiary Hospital, Bhutan.

Factors	cOR (95% CI)	*p*‐value	aOR (95% CI)	*p*‐value
**Age**				
> 40 (Ref)				
< 21	0.3 (0.18–0.68)	0.002	0.4 (0.14–1.25)	0.119
21–40	0.6 (0.34–0.94)	0.027	0.9 (0.51–1.47)	0.583
**Gender**				
Female (Ref)				
Male	2.3 (1.27–4.11)	0.006	1.8 (0.89–3.44)	0.103
**Visit type**				
OPD (Ref)				
IPD	3.7 (2.30–5.87)	< 0.001	2.4 (1.34–4.37)	0.003
**Department**				
Surgery (Ref)				
Emergency	0.9 (0.40–2.07)	0.826	0.7 (0.31–1.56)	0.493
General practice	0.3 (0.13–0.68)	0.007	0.5 (0.20–1.20)	0.120
Obstetrics & gynecology	0.4 (0.17–1.99)	0.071	0.7 (0.25–1.74)	0.402
Medicine	1.8 (0.66–4.93)	0.099	1.5 (0.53–4.16)	0.459
Pediatric	0.3 (0.09–0.78)	0.016	0.7 (0.14–3.24)	0.630

Abbreviations: aOR, adjusted odds ratio; CI, confidence interval; cOR, crude odds ratio; IPD, inpatient department; OPD, outpatient department; Ref, reference.

## 4 Discussion

A rising trend of antimicrobial resistance among uropathogens poses a burden worldwide.While international initiatives increasingly emphasize antimicrobial stewardship as a primary intervention, the effective implementation of these programs requires a clear understanding of the baseline resistance burden. Therefore, our study evaluated the antimicrobial susceptibility patterns and proportion of multidrug‐resistant uropathogens.

In the current study, Gram‐negative uropathogens were predominant, accounting for over 98% of the isolates. Among Gram‐negative uropathogens, the most common was *E. coli* (80.6%), followed by *Klebsiella pneumoniae* (15.6%) – which is consistent with findings from regional and global studies [7, 17, 26, 27, 28, 29, 30, 31]. Notably, cross‐tabulation revealed that this microbial isolate distribution remained statistically uniform across all age categories (*p* = 0.590) and between sex groups (*p *= 0.145) (Supplementary Tables 2 and 3). In contrast, international studies report significant declines in *E. coli* predominance among older adults and males [32, 33]. Although a higher numerical recovery of *Klebsiella pneumoniae* was observed in males (20.8% vs. 14.4%) – a pattern reported in other cohorts [32], this subgroup difference did not reach statistical significance in our sample. Altogether, this finding reaffirms that the majority of the uropathogens are Gram‐negative organisms globally.

Antibiotics are classified as ‘Access,' ‘Watch,' and ‘Reserve' groups by WHO based on the clinical importance and the risk of their use promoting resistances [25]. According to the antimicrobial susceptibility pattern, *E. coli* was highly susceptible to Access group of antibiotics ‐gentamicin (81.1%), followed by amikacin (78.3), nitrofurantoin (71.2%) and sulfamethoxazole‐trimethoprim (67.9%), aligning with the findings of studies conducted in Nepal and India [34, 35]. However, it exhibited lower susceptibility to Access group ‐nitrofurantoin, compared to European outpatient data [31]. Similarly, *Klebsiella pneumoniae* isolates demonstrated high sensitivity to Access group‐gentamicin resembling pattern from Nepal and India [7, 35]. For Watch group antibiotics, *E. coli* mirrors pattern across Asia with moderate‐to‐high susceptibility to imipenem and meropenem [7, 9, 11, 17, 26]. This is likely due to restricted access and unavailability as empirical treatment in our setting, while showing significant resistance to ceftriaxone, consistent with acute emergency care data in Türkiye [30]. Furthermore, *Klebsiella pneumoniae* showed high susceptibility to Watch group‐fluoroquinolone similar to studies from Nepal, whereas studies from Pakistan and Türkiye reported minimal susceptibility [7, 11, 30]. Variations in antimicrobial susceptibility patterns across countries could reflect differences in antibiotic use, availability of antibiotics, prescribing practices, lack of quality diagnostic facilities, poor compliance, poor sanitation and hygiene and local epidemiological factors. Additionally, cross‐study comparisons must be interpreted with caution due to differing breakpoint definitions; following CLSI guidelines, we reported Intermediate (‘I') isolates as a standalone category for baseline susceptibility, whereas EUCAST‐aligned European studies categorize ‘I' as ‘Susceptible, increased exposure' and group them with susceptible strains.

The emerging of MDRO poses a significant public health concern with a major threat to patient safety and healthcare quality. Approximately two‐fifth (39.2%) of the positive urine cultures in our study were MDRO with CRO accounting for accounting for 8.2% of these resistant isolates. However, the total proportion of MDRO was low compared to Nepal (54%), Bangladesh (71.19%), and Pakistan (47.3%), while the proportion of CRO is also low compared to reports from Türkiye (17.9%) and India (22.6%) [20, 30, 36, 37, 38]. The relatively lower burden of the MDRO and CRO could be partly influenced by the contextual factors unique to the region. The geographical isolation of the hospital from densely populated urban centers limits the introduction of highly resistant organism. Moreover, strict regulation of antibiotic sale and distribution by the Bhutan Food and Drug Authority reduces inappropriate self‐medication which is common cause of the resistance [39]. Furthermore, limited cross‐border patient movement contributes to minimal influx and transmission. Despite CRO being reported at relatively low levels overall, their presence itself is clinically significant as these strains represent high‐level antimicrobial resistance, which is inherently associated with high rates of mortality, strong potential for rapid transmission, and a growing role as an emerging cause of healthcare‐associated infections (20). On multivariable logistic regression analysis, inpatient status remained the sole independent predictor significantly associated with MDRO isolation (aOR 2.4, 95% CI 1.34–4.37, *p* = 0.003), reinforcing evidence of similar epidemiological patterns across South Asia especially in Nepal and Bangladesh [20, 40] The substantially lower susceptibility to several Access and Watch group antibiotics observed among IPD isolates (Supplementary Table 1) aligns with IPD status being the sole predictor of MDRO, likely reflecting multiple prior antibiotic exposure. This higher risk in hospitalized patients could be also due to underlying medical conditions and indwelling medical device such as urinary catheter and cannula.

In present study, positive culture was predominant in female (85.8%). Similar results have been widely reported from Bangladesh (71.2%), Afghanistan (59.8%), Nepal (69.5%) and Pakistan (59.6%) [7, 9, 11, 17]. The higher prevalence of UTI among female compared to male could be due to anatomical and physiological factors such as a shorter and wider urethra, the adjacency of urethral opening to the anus and hormonal influences [7, 41].

The urine culture positivity rate observed in our study was around 20%, which is similar with the studies from India (22.9%) and Nepal (22.2%) [27, 34] However, a higher positive rate was reported from Pakistan (42.6%), Afghanistan (65.9%) and European cohort such as France (75.2%) [11, 17, 31]. These variations maybe heavily influenced by differences in patient selection criteria and clinical settings across studies; while higher‐yield reports often enrolled pre‐screened, and clinically symptomatic patients, our study evaluated broad, unselected urine culture requests across routine clinical encounters. Additionally, a lower positive rate in our study could be partly due to physicians initiating empirical antibiotic treatment based on routine urine examination report, in absence of culture and sensitivity report. This might have contributed to culture negative results despite inappropriate treatment.

### 4.1 Strengths and Limitations

Strengths of the study include the inclusion all eligible culture‐positive urine samples generated over a complete calendar year, providing a comprehensive local snapshot of uropathogen distribution and resistance and application of WHO AWaRE framework. The study also provides data from a geographically underrepresented remote tertiary hospital in Bhutan, where local antibiogram data are limited. Despite its substantial insights, this cross‐sectional study has several limitations. First, the retrospective design limits clinical information, such as comorbidities, prior antibiotic exposure, and the presence of indwelling medical devices. Because clinical symptoms could not be evaluated, positive urine cultures could represent asymptomatic bacteriuria or colonization rather than active, clinically confirmed urinary tract infections. Furthermore, it was not possible to distinguish catheter‐associated urinary tract infections from community‐acquired or non‐catheterized cases, requiring culture positivity to be interpreted uniformly using general laboratory criteria. Second, the study was conducted at a single tertiary hospital, limiting generalizability to other settings. Third, specific laboratory constraints affected antimicrobial testing. The laboratory used a tiered antimicrobial susceptibility testing strategy with second‐line agents tested selectively; therefore, susceptibility estimates for these agents were based on smaller, selected denominators and should not be interpreted as population‐wide estimates. Crucially, because only seven antimicrobial classes were available in our setting rather than the full ECDC/CDC consensus panel – advanced resistance profiles could only be classified as p‐XDR and p‐PDR. Consequently, these p‐XDR and p‐PDR counts reflect local panel limits rather than true, proven resistance, making direct comparisons with global studies difficult. Additionally, molecular testing facilities were unavailable to determine the specific genetic resistance mechanisms behind the observed phenotypic MDRO and CRO patterns. Finally, due to local resource constraints, phenotypic confirmatory testing for extended‐spectrum beta‐lactamase (ESBL) production and carbapenemase activity was not routinely performed, precluding the reporting of definitive ESBL positivity rates and enzymatic carbapenemase mechanisms.

### 4.2 Recommendations

The findings from this study provide important epidemiological data to inform local clinical practice and future research. Based on the observed antimicrobial susceptibility, Access group antibiotics ‐ nitrofurantoin and gentamicin demonstrated relatively preserved susceptibility. However, as an empirical antimicrobial selection requires strict individualization based on the anatomical site of infection, clinical severity, patient comorbidities, and renal function. Specifically, nitrofurantoin is appropriate only for lower, uncomplicated UTIs and has key limitations in patients with impaired renal clearance, whereas empirical use of gentamicin requires consideration of regional resistance baseline and careful risk assessment due to potential nephrotoxicity and ototoxicity. The presence of MDRO and CRO in our study underscores the urgent need to strengthen infection prevention and control practices, and to improve antimicrobial stewardship programs. In addition, there is an urgent need to develop national and or hospital antibiotic guideline, extension of culture and sensitivity testing facilities to other health centers and need for continuous surveillance. In view of significant proportion of UTI among female, further prospective study is required to explore the risk factors among women to help develop preventive strategies and reduce the burden of infection.

## 5 Conclusion

Culture positive rate was approximately 20%, with majority caused by Gram‐negative bacteria‐*E. coli*, demonstrating high susceptibility to Access group of antibiotics. Approximately two‐fifth of the uropathogens were MDRO‐which highlights the growing concern of antimicrobial resistance.

The findings underscore the need of consistent local surveillance, local antibiogram, rational antibiotic prescribing practice, stringent infection prevention and control practice and robust antimicrobial stewardship programs.

## Author Contributions

**Sanga Tenzin:** conceptualization, methodology, investigation, validation, formal analysis, supervision, data curation, visualization, writing – original draft, writing – review and editing, project administration, resources. **Yeshey Dorjey:** conceptualization, methodology, data curation, investigation, validation, formal analysis, supervision, visualization, writing – original draft, writing – review and editing, resources, project administration. **Pelden Wangchuk:** supervision, writing – original draft, writing – review and editing, project administration. **Chimi Wangmo:** conceptualization, validation, project administration, data curation, writing – review and editing. **Nima Dorji:** conceptualization, methodology, project administration, data curation, writing – review and editing. **Ugyen Rinzin:** conceptualization, writing – original draft, writing – review and editing, validation, project administration, data curation, resources. **Ugyen Chenzom:** conceptualization, writing – review and editing, formal analysis, data curation, resources. **Tsheten Wangchuk:** conceptualization, writing – original draft, writing – review and editing, methodology, project administration, resources, data curation.

## Funding

The authors have nothing to report.

## Ethics Statement

Administrative clearance was obtained from the Ministry of Health, and waiver of ethical clearance was granted by the Institutional Review Board, Khesar Gyelpo University of Medical Sciences of Bhutan, with Ref. No. IRB/Waiver‐Exempt/PN/2025/064/114, dated 4th December 2025.

## Conflicts of Interest

The authors declare no conflicts of interest.

## Transparency Statement

Mr. Sanga Tenzin affirms that this manuscript is an honest, accurate, and transparent account of the study being reported; that no important aspects of the study have been omitted; and that any discrepancies from the study as planned (and, if relevant, registered) have been explained.

## Supporting information


Supporting File 1

Supporting File 2

## Data Availability

The data that support the findings of this study are available from the corresponding author upon reasonable request.
