# Supplementary material for: Antimicrobial Susceptibility Patterns and Proportion of Multidrug‐Resistant Uropathogens in a Remote Tertiary Hospital in Bhutan: A Cross‐Sectional Study
Source: Health Sci Rep. 2026 Oct 7;9(10):e73364. doi: 10.1002/hsr2.73364 (PMC13646896; doi:10.1002/hsr2.73364)
Supplement: Supplementary file 1 [file HSR2-9-e73364-s001.docx]

**Supplementary Table 2: Distribution of Microbial isolates across Age interval at a remote tertiary hospital, Bhutan**

| **Microbial Isolate** | **Total,**  **n = 372** | **<21 years,**  **n = 56** | **21–40 years,**  **n = 110** | **>40 years,**  **n = 206** | ***p-value*** |
| --- | --- | --- | --- | --- | --- |
| *E. coli* | 295 (79.3) | 47 (83.9) | 88 (80.0) | 160 (77.7) | 0.590 |
| *Klebsiella pneumoniae* | 57 (15.3) | 9 (16.1) | 15 (13.6) | 33 (16.0) |  |
| *Pseudomonas* spp*.* | 11 (3.0) | 0 (0.0) | 3 (2.7) | 8 (3.9) |  |
| *Staphylococcus spp.* | 3 (0.8) | 0 (0.0) | 2 (1.8) | 1 (0.5) |  |
| *Streptococcus* spp*.* | 3 (0.8) | 0 (0.0) | 0 (0.0) | 3 (1.5) |  |
| *Citrobacter* spp. | 2 (0.5) | 0 (0.0) | 1 (0.9) | 1 (0.5) |  |
| *Proteus* spp. | 1 (0.3) | 0 (0.0) | 1 (0.9) | 0 (0.0) |  |

**Supplementary Table 3: Distribution of Microbial isolates by Gender at a remote tertiary hospital, Bhutan**

| **Microbial Isolate** | **Total, n = 372** | **Female, n = 319** | **Male, n = 53** | ***p-value*** |
| --- | --- | --- | --- | --- |
| *E. coli* | 295 (79.3) | 258 (80.9) | 37 (69.8) | 0.145 |
| *Klebsiella pneumoniea* | 57 (15.3) | 46 (14.4) | 11 (20.8) |  |
| *Pseudomonas* spp. | 11 (3.0) | 7 (2.2) | 4 (7.5) |  |
| *Staphylococcus spp.* | 3 (0.8) | 3 (0.9) | 0 (0.0) |  |
| *Streptococcus* spp. | 3 (0.8) | 3 (0.9) | 0 (0.0) |  |
| *Citrobacter* spp. | 2 (0.5) | 1 (0.3) | 1 (1.9) |  |
| *Proteus* spp. | 1 (0.3) | 1 (0.3) | 0 (0.0) |  |

**Supplementary Table 4: Antimicrobial susceptibility profiles of isolated uropathogens categorized by drug class at a remote tertiary hospital, Bhutan**

| **Antibiotic Class** | **Antibiotics** | | ***E. coli***  ***n/N*** | ***Klebsiella pneumoniae n/N*** | ***Pseudomonas* spp. n/N** | ***Citrobacter* spp*. n/N*** | ***Proteus* spp*. n/N*** | ***Staphylococcus saprophyticus n/N*** | ***Streptococcus* spp. n/N** |
| --- | --- | --- | --- | --- | --- | --- | --- | --- | --- |
| **Aminoglycosides** | **Gentamicin** | |  |  |  |  |  |  |  |
|  |  | S | 236/291 (81.1) | 51/54 (94.4) | 9/11 (81.8) | 2/2 (100.0) | 1/1 (100.0) | x | x |
|  |  | NS | 55/291 (18.9) | 3/54 (5.6) | 2/11 (18.2) | 0/2 (0.0) | 0/1 (0.0) | x | x |
|  | **Amikacin** | |  |  |  |  |  |  |  |
|  |  | S | 18/23 (78.3) | 3/5 (60.0) | 5/6 (83.3) | x | x | x | x |
|  |  | NS | 5/23 (21.7) | 2/5 (40.0) | 1/6 (16.7) | x | x | x | x |
| **Penicillin** | **Ampicillin** | |  |  |  |  |  |  |  |
|  |  | S | 128/293 (43.7) | 0/55 (0.0) | x | 0/2 (0.0) | 1/1 (100.0) | x | x |
|  |  | NS | 165/293 (56.3) | 55/55 (100.0) | x | 2/2 (100.0) | 0/1 (0.0) | x | x |
|  | **Penicillin G** | |  |  |  |  |  |  |  |
|  |  | S | x | x | x | x | x | 1/3 (33.3) | 3/3 (100.0) |
|  |  | NS | x | x | x | x | x | 2/3 (66.7) | 0/3 (0.0) |
|  | **Piperacillin** | |  |  |  |  |  |  |  |
|  |  | S | x | x | 7/9 (77.8) | x | x | x | x |
|  |  | NS | x | x | 2/9 (22.2) | x | x | x | x |
| **Cephalosporins** | **Cefazolin** | |  |  |  |  |  |  |  |
|  |  | S | 167/291 (57.4) | 31/55 (56.4) | x | 0/2 (0.0) | 1/1 (100.0) | x | x |
|  |  | NS | 124/291 (42.6) | 24/55 (43.6) | x | 2/2 (100.0) | 0/1 (0.0) | x | x |
|  | **Ceftriaxone** | |  |  |  |  |  |  |  |
|  |  | S | 162/290 (55.9) | 31/55 (56.4) | x | 1/2 (50.0) | 1/1 (100.0) | x | x |
|  |  | NS | 128/290 (44.1) | 24/55 (43.6) | x | 1/2 (50.0) | 0/1 (0.0) | x | x |
|  | **Ceftazidime** | |  |  |  |  |  |  |  |
|  |  | S | x | x | 5/7 (71.4) | x | x | x | x |
|  |  | NS | x | x | 2/7 (28.6) | x | x | x | x |
| **Fluoroquinolones** | **Norfloxacin** | |  |  |  |  |  |  |  |
|  |  | S | 177/287 (61.7) | 46/54 (85.2) | 9/10 (90.0) | 1/2 (50.0) | 1/1 (100.0) | 3/3 (100.0) | x |
|  |  | NS | 110/287 (38.3) | 8/54 (14.8) | 1/10 (10.0) | 1/2 (50.0) | 0/1 (0.0) | 0/3 (0.0) | x |
| **Nitrofuran** | **Nitrofurantoin** | |  |  |  |  |  |  |  |
|  |  | S | 208/292 (71.2) | 10/54 (18.5) | x | 1/2 (50.0) | x | 2/3 (66.7) | x |
|  |  | NS | 84/292 (28.8) | 44/54 (81.5) | x | 1/2 (50.0) | x | 1/3 (33.3) | x |
| **Folate pathway inhibitors** | **Sulphamethoxazole-Trimethoprim** | |  |  |  |  |  |  |  |
|  |  | S | 197/290 (67.9) | 33/55 (60.0) | x | 1/2 (50.0) | x | 3/3 (100.0) | x |
|  |  | NS | 93/290 (32.1) | 22/55 (40.0) | x | 1/2 (50.0) | x | 0/3 (0.0) | x |
| **Carbapenems** | **Imipenem** | |  |  |  |  |  |  |  |
|  |  | S | 16/19 (84.2) | 2/4 (50.0) | 0/1 (0.0) | x | x | x | x |
|  |  | NS | 3/19 (15.8) | 2/4 (50.0) | 1/1 (100.0) | x | x | x | x |
|  | **Meropenem** | |  |  |  |  |  |  |  |
|  |  | S | 18/24 (75.0) | 1/5 (20.0) | 0/1 (0.0) | x | x | x | x |
|  |  | NS | 6/24 (25.0) | 4/5 (80.0) | 1/1 (100.0) | x | x | x | x |

*NS: non- susceptible; S: susceptible; n/N: represents the exact count of isolates categorized as S or NS and ‘N’- represents the total number of isolates tested for that specific antibiotic-organism; x: not tested; ; NS represents a pooled analytical category (Intermediate + Resistant)*
