# Supplementary material for: Antimicrobial Susceptibility Patterns and Proportion of Multidrug‐Resistant Uropathogens in a Remote Tertiary Hospital in Bhutan: A Cross‐Sectional Study
Source: Health Sci Rep. 2026 Oct 7;9(10):e73364. doi: 10.1002/hsr2.73364 (PMC13646896; doi:10.1002/hsr2.73364)
Supplement: Supplementary file 2 [file HSR2-9-e73364-s002.docx]

| **Supplementary Table 1: Comparison of antibiotic susceptibility between Outpatient and Inpatient Department, at a remote Tertiary Hospital, Bhutan** | | | | | |  |
| --- | --- | --- | --- | --- | --- | --- |
| **Antibiotics, n (%)** | | **Total** | **OPD** | **IPD** | ***p*- vlaue** | |
| **ACCESS GROUP** | | | | | | |
| Ampicillin (n=294) | |  |  |  |  | |
|  | S | 129 (43.9) | 80 (62.0) | 49 (38.0) | <0.001 | |
|  | I | 8 (2.7) | 3 (37.50 | 5 (62.5) |  | |
|  | R | 157 (53.4) | 47 (29.9) | 110 (70.1) |  | |
| Penicillin G (n=8) | |  |  |  |  | |
|  | S | 4 (50.0) | 1 (25.0) | 3 (75.0) | 0.909 | |
|  | I | 2 (25.0) | 1 (50.0) | 1 (50.0) |  | |
|  | R | 2 (25.0) | 1 (50.0) | 1 (50.0) |  | |
| Cefazolin (n=347) | |  |  |  |  | |
|  | S | 199 (57.3) | 113 (56.8) | 86 (43.2) | <0.001 | |
|  | I | 8 (2.3) | 2 (25.0) | 6 (75.0) |  | |
|  | R | 140 (40.4) | 30 (21.4) | 110 (78.6) |  | |
| Amikacin (n=34) | |  |  |  |  | |
|  | S | 26 (76.5) | 4 (15.4) | 22 (84.6) | 0.004 | |
|  | I | 2 (5.9) | 0 (0.0) | 2 (100) |  | |
|  | R | 6 (17.6) | 0 (0.0) | 6 (100) |  | |
| Gentamicin (n=359) | |  |  |  |  | |
|  | S | 299 (83.3) | 129 (43.1) | 170 (56.9) | 0.129 | |
|  | I | 7 (1.9) | 2 (28.6) | 5 (71.4) |  | |
|  | R | 53 (14.8) | 14 (26.4) | 39 (73.6) |  | |
| Nitrofurantoin (n=351) | |  |  |  |  | |
|  | S | 221 (63.0) | 101 (45.7) | 120 (54.3) | 0.003 | |
|  | I | 32 (9.1) | 15 (46.9) | 17 (53.1) |  | |
|  | R | 98 (27.9) | 32 (32.7) | 66 (67.3) |  | |
| Suphamethoxazole-trimethprim (n=350) | |  |  |  |  | |
|  | S | 234 (66.9) | 117 (50.0) | 117 (50.0) | <0.001 | |
|  | I | 3 (0.9) | 1 (33.3) | 2 (66.7) |  | |
|  | R | 113 (33.3) | 29 (25.7) | 84 (74.3) |  | |
| **WATCH GROUP** | | | | | | |
| Piperacillin (n=9) | |  |  |  |  | |
|  | S | 7 (77.8) | 0 (0) | 7 (100) | 0.044 | |
|  | I | 0 (0.0) | 0 (0) | 0 (0) |  | |
|  | R | 2 (22.2) | 0 (0) | 2 (100) |  | |
| Ceftazidime (n=7) | |  |  |  |  | |
|  | S | 5 (71.4) | 0 (0) | 5 (100) | 0.09 | |
|  | I | 0 (0.0) | 0 (0) | 0 (0) |  | |
|  | R | 2 (28.6) | 0 (0) | 2 (100 |  | |
| Ceftriaxone (n=348) | |  |  |  |  | |
|  | S | 195 (56.0) | 111 (56.9) | 84 (43.1) | <0.001 | |
|  | I | 6 (1.7) | 2 (33.3) | 4 (66.7) |  | |
|  | R | 147 (42.2) | 33 (22.4) | 114 (77.6) |  | |
| Norfloxacin (N=357) | |  |  |  |  | |
|  | S | 237 (66.4) | 114 (48.1) | 123 (51.9) | 0.001 | |
|  | I | 5 (1.4) | 2 (40.0) | 3 (60.0) |  | |
|  | R | 115 (32.2) | 30 (26.1) | 85 (73.9) |  | |
| Imipenem (n=24) | |  |  |  |  | |
|  | S | 18 (75.0) | 3 (16.7) | 15 (83.3) | 0.032 | |
|  | I | 2 (8.3) | 0 (0.0) | 2 (100) |  | |
|  | R | 4 (16.7) | 0 (0.0) | 4 (100) |  | |
| Meropenem (n=30) | |  |  |  |  | |
|  | S | 19 (63.3) | 3 (15.8) | 16 (84.20 | 0.001 | |
|  | I | 0 (0.0) | 0 (0.0) | 0 (0.0) |  | |
|  | R | 11 (36.7) | 0 (0.0) | 11 (100) |  | |

*IPD: inpatient department; I: intermediate; OPD: outpatient department; R: resistant; S: susceptible*
